# Comparing Proxy, Adolescent, and Adult Assessments of Functional Ability in Adolescents With Juvenile Idiopathic Arthritis

**DOI:** 10.1002/acr.23877

**Published:** 2020-03-27

**Authors:** Stephanie J. W. Shoop‐Worrall, Kimme L. Hyrich, Suzanne M. M. Verstappen, Jamie C. Sergeant, Eileen Baildam, Alice Chieng, Joyce Davidson, Helen Foster, Yiannis Ioannou, Flora McErlane, Lucy R. Wedderburn, Wendy Thomson, Janet E. McDonagh

**Affiliations:** ^1^ University of Manchester Manchester UK; ^2^ Central Manchester University Hospitals NHS Foundation Trust Manchester Academic Health Science Centre and University of Manchester Manchester UK; ^3^ University of Manchester and Central Manchester University Hospitals NHS Foundation Trust Manchester Academic Health Science Centre Manchester UK; ^4^ Alder Hey Children’s NHS Foundation Trust Liverpool UK; ^5^ Royal Manchester Children’s Hospital Manchester UK; ^6^ Royal Hospital for Children, Glasgow, UK and Royal Hospital for Sick Children Edinburgh UK; ^7^ Great North Children’s Hospital Newcastle Hospitals NHS Foundation Trust and Newcastle University Newcastle upon Tyne UK; ^8^ University College London London UK; ^9^ Great North Children’s Hospital Newcastle Hospitals NHS Foundation Trust Newcastle upon Tyne UK; ^10^ University College London Great Ormond Street Hospital NHS Foundation Trust and NIHR Great Ormond Street Hospital Biomedical Research Centre London UK; ^11^ Present address: UCB Celltech ‎Slough UK

## Abstract

**Objective:**

In pediatric research, investigators rely on proxy reports of outcome, such as the proxy‐completed Childhood Health Assessment Questionnaire (C‐HAQ), to assess function in juvenile idiopathic arthritis (JIA). As children mature, they may self‐complete the adult HAQ or the unvalidated adolescent‐specific C‐HAQ. It is unclear how these measures compare and whether they are directly interchangeable. The present study was undertaken to compare agreement between the proxy‐completed C‐HAQ, adolescent‐specific C‐HAQ, and the HAQ at initial presentation to pediatric rheumatologic care and 1 year following the first presentation in adolescents with JIA.

**Methods:**

Adolescents ages 11–17 years participating in the Childhood Arthritis Prospective Study (CAPS), a UK multicenter inception cohort, were included. In a CAPS substudy, adolescents self‐completed the adolescent‐specific C‐HAQ and the HAQ, and proxies simultaneously completed the proxy‐completed C‐HAQ at baseline and 1 year. Correlation and agreement between scores were assessed at baseline. Agreement and ability to similarly classify clinically important changes over time were assessed at 1 year following initial presentation to rheumatologic care.

**Results:**

A total of 107 adolescents (adolescent‐specific C‐HAQ and HAQ) or their proxies (proxy‐completed C‐HAQ) had completed all 3 measures at baseline. Median age at diagnosis was 13 years, and 61% were female. Although the 3 scores demonstrated strong correlations (r > 0.8), they were not completely interchangeable, with agreement ranging between 70% and 80%. There was similar agreement between the changes in scores between baseline and 1 year. Using proxy‐completed C‐HAQ minimum clinically important cutoffs, the adolescent‐specific C‐HAQ and the HAQ similarly classified 80% to 90% of adolescents as having improved or worsened.

**Conclusion:**

While there is relatively high agreement and similar classification of change between HAQ and the 2 C‐HAQ scores, these are not completely interchangeable. This impacts the comparison of function when measured in different ways over the lifespan.

## Introduction

One of the main challenges in adolescent medicine is the continuous monitoring of disease activity and its impact on psychosocial outcomes as the young person develops physically, socially, and cognitively. It is during this time that adolescents will be moved from child‐ to adult‐centered rheumatology services, rendering continuous data capture over this period even more challenging 1.

**Significance & Innovations**

- The proxy‐completed Childhood Health Assessment Questionnaire (C‐HAQ), adolescent‐specific C‐HAQ, and adult HAQ are highly correlated and have moderate‐to‐high agreement in young people with juvenile idiopathic arthritis (JIA). This means that the adolescent‐specific C‐HAQ and the HAQ are both suitable outcome measures for adolescents with JIA.
- Both C‐HAQ scores consistently exceed HAQ scores. These scores are therefore not completely interchangeable. This may affect the longer‐term study of function over time as young people get older within pediatric practice and through the transfer process to adult services.

In pediatric rheumatology, both younger children (age <11 years) and adolescents (ages 11–19 years) with juvenile idiopathic arthritis (JIA) are often monitored using similar outcomes. In accordance, the core outcome variables for JIA are captured across this spectrum 2. However, the measures used to capture these outcomes may not always be developmentally appropriate for the adolescent age range.

Functional ability is one such core outcome variable in JIA 2. The most commonly used measure of functional ability in JIA is the proxy‐completed Childhood Health Assessment Questionnaire (C‐HAQ) 3. The proxy‐completed C‐HAQ is a questionnaire written in the third person comprising 30 questions corresponding to 8 domains of functional ability: dressing and grooming, arising, eating, walking, hygiene, reach, grip, and activities. Lower overall scores denote better functional ability, with multiple cutoffs between 0.13 and 0.75 (range 0–3) denoting mild‐to‐moderate functional changes 4, 5. Although scores along the entire length of the scale are possible, the C‐HAQ has a known flooring effect, with scores clustering to the lower (better) end of the scale 4.

While the proxy‐completed C‐HAQ has been validated for use in children and young people with JIA up to the ages of 19 years 3, it is written in the third person and hence does not convey the importance of the perspective of the young person and particularly their need for increasing confidentiality and privacy as they go through adolescence 6, 7. Furthermore, some of the terms in the UK version are not developmentally appropriate for adolescents (such as the ability to use a potty or tricycle), rending the questionnaire unpopular in this age group 3, 7. To combat these issues, an adolescent‐specific version of the C‐HAQ was developed in 2006 6. This measure was designed to be self‐completed, with rewording and altered outcome categories included. While the proxy‐completed C‐HAQ and the adolescent‐specific C‐HAQ have shown excellent agreement with each other 7, the latter has not been formally validated. The addition of a separate outcome measure for adolescents also hinders the continuous capture of similarly measured outcome data over time. A single measure to capture functional ability through adolescence and adulthood would be preferable, or alternatively the ability to directly compare underlying function across questionnaires or scoring within an individual.

The HAQ, designed to measure functional ability in adults (including young individuals age 18 years or older) with rheumatologic conditions such as rheumatoid arthritis 8, is used in adult rheumatology clinics that are attended by adults with JIA. The proxy‐completed C‐HAQ was originally validated against this measure, which contains the same domains but has fewer questions and is designed for proxy completion 3. Use of the HAQ in adolescents with JIA through adulthood would aid in the continuous capture of this important outcome measure. However, to date, no study has directly compared the proxy‐completed C‐HAQ, the adolescent‐specific C‐HAQ, and the HAQ in a single population. The aims of the current study were to compare agreement between the proxy‐completed C‐HAQ, adolescent‐specific C‐HAQ, and the HAQ at initial presentation to pediatric rheumatologic care and 1 year following first presentation in adolescents with JIA.

## Patients and Methods

#### Study population

Adolescents with JIA were selected from the Childhood Arthritis Prospective Study (CAPS). CAPS is a prospective, multicenter inception cohort in the UK following children and young people with inflammatory arthritis with onset before their 16th birthday. Specific inclusion and exclusion criteria for CAPS have been described previously 9. CAPS was approved by the Northwest Multicentre Research Ethics Committee (REC/02/8/104, IRAS 184042), and written informed consent was provided by proxies (parents, caregivers, legal guardians) for all participants. Where possible, assent was also obtained.

Between January 2004 and January 2015, as a substudy, adolescents enrolled in CAPS who were ages 11–17 years at the point of first appointment with a pediatric or adolescent rheumatologist were asked to self‐complete the adolescent‐specific C‐HAQ and the HAQ; their proxies were asked to complete the proxy‐completed C‐HAQ simultaneously. Only those adolescents with complete data on all 3 questionnaires at initial presentation to pediatric rheumatologic care (no specific requirement for 1 year data availability) were included in this study.

#### Data collection.

##### Functional ability questionnaires

Proxies completed the proxy‐completed C‐HAQ at baseline and at the 1‐year follow‐up. The adolescent‐specific C‐HAQ and HAQ scores were self‐completed by the adolescents at these time points. Although all 3 questionnaires comprise questions covering the same 8 domains of functional ability, there are distinct differences between the forms. Where the proxy‐completed C‐HAQ is designed for proxy completion, the adolescent‐specific C‐HAQ and HAQ are worded as self‐completed questionnaires. The proxy‐completed C‐HAQ and adolescent‐specific C‐HAQ both comprise 30 questions, although developmentally inappropriate terms for adolescents such as “potty” and “tricycle” and the not applicable column for developmentally inappropriate activities for young children (e.g., opening car doors) have been removed on the adolescent‐specific C‐HAQ (see Supplementary Table 1, available on the *Arthritis Care & Research* web site at http://onlinelibrary.wiley.com/doi/10.1002/acr.23877/abstract).

##### Calculating scores for the proxy‐completed C‐HAQ, the adolescent‐specific C‐HAQ, and the HAQ

Although the HAQ has 10 fewer questions than the C‐HAQ questionnaires and the wording is adult‐centered (e.g., “Reach and get down a 5‐lb object [e.g., a bag of potatoes]” in the HAQ versus “Reach and get down a heavy object such as a large game or books” in the C‐HAQ), the scoring system across all 3 questionnaires is identical. The score for each domain is the highest score (0–3 scale) from any question within that section. If the use of any aids has been indicated for a specific domain and the current score is <2, that domain‐specific score is raised to 2. For all questionnaires, if at least 6 of the 8 domains have been scored, total scores are divided by the number of domains completed, for a final score on a 0–3 scale (with a score of 3 indicating the worst functional ability).

The final scores (and item scores, where provided) on the 3 measures were recorded in the study databases. Where item scores were provided, final scores were calculated by the investigators. If component scores were not provided (n = 5), total scores calculated by recruiting center staff were used.

#### Statistical analysis

Initially, medians and interquartile ranges (IQRs) were compared descriptively between baseline scores using Wilcoxon's signed rank tests to assess statistical significance. The primary analysis was agreement between overall scores on the 3 functional ability measures at initial presentation to pediatric rheumatologic care. Agreement was assessed graphically using Bland‐Altman plots with 95% limits of agreement 10 and through assessing the percentage of scores within ±0.25 points, as has previously been assessed 6.

Secondary analyses included correlations between pairs of baseline total (Spearman's correlation) and domain‐specific scores (Cohen's linear‐weighted kappa coefficients) because scores were not normally distributed. Correlation coefficients were considered strong if exceeding 0.7 11. Kappa coefficients indicated moderate agreement if exceeding 0.4 and substantial if exceeding 0.6 12. In addition, changes in overall scores between baseline and 1 year were assessed in complete case analyses. Agreement between these changes was compared between pairs of function questionnaires via Bland‐Altman plots. Where the HAQ and adolescent‐specific C‐HAQ are considered equivalent to the proxy‐completed C‐HAQ, similar minimum clinically important differences in score could therefore be considered applicable across all 3 measures. Published minimum clinically important improvements (–0.188) and worsening (+0.125) from the proxy‐completed C‐HAQ 13 were therefore tested on the HAQ and adolescent‐specific C‐HAQ to assess the similarity of classification. These cut points were applied to changes in adolescent‐specific C‐HAQ and HAQ scores for 2 dichotomous variables per questionnaire: improved (y/n) and worsened (y/n). Since 0.188 could not be scored directly on the C‐HAQ/HAQ, which improve in units of 0.125, improvements of 0.125 and 0.25 were tested. Area under the curve (AUC) analyses using receiver operating characteristics then determined the percentage of adolescents similarly classified as having improved or worsened on the adolescent‐specific C‐HAQ or HAQ compared with the proxy‐completed C‐HAQ. Optimal cut points were then determined for the adolescent‐specific C‐HAQ and the HAQ that would lead to the largest AUC for improved or worsened scores compared with the proxy‐completed C‐HAQ.

## Results

#### Patient cohort

A total of 451 adolescents ages 11–17 years were recruited to CAPS between January 2004 and January 2015. Of these, 104 had complete data on the 3 measures, i.e., proxy‐completed C‐HAQ, adolescent‐specific C‐HAQ, and the HAQ, at baseline, and 92 adolescents had data on at least 2 of the questionnaires at baseline and 1 year (n = 70 for the proxy‐completed C‐HAQ and adolescent‐specific C‐HAQ measures, n = 67 for the proxy‐completed C‐HAQ and HAQ measures, and n = 65 for the adolescent‐specific C‐HAQ and HAQ measures). Adolescents with incomplete function data were no different than those with complete function data in terms of the demographic or disease activity levels described in Table 1. The majority were female (61%) and of white ethnicity (89%), and the most common International League of Associations for Rheumatology (ILAR) category was oligoarticular JIA (44%). Median age at initial presentation to pediatric rheumatologic care was 13 years (IQR 12–14 years), with a median time between symptom onset and initial presentation to pediatric rheumatologic care of 7 months (IQR 5–14 months). Adolescents had a median of 2 active joints (IQR 1–5) and median scores on both the physician and parent global assessments of 2.5 cm (Table 1).

**Table 1:** Baseline characteristics of the cohort^a^

Characteristic	Complete data, %	Value
Female sex	100	63 (61)
White race	97	90 (89)
Age at disease onset, median (IQR) years	95	12 (11–14)
Age at first presentation, median(IQR) years	100	13 (12–14)
Symptom duration at first pediatric rheumatology appointment,median (IQR) months	95	7 (5–14)
ILAR category	100	
Systemic		8 (7)
Oligoarticular		46 (44)
RF‐negative polyarticular		16 (15)
RF‐positive polyarticular		4 (4)
Enthesitis‐related		5 (5)
Psoriatic		14 (13)
Undifferentiated		12 (11)
Core outcome variables at baseline		
Active joint count, median (IQR)	89	2 (1–5)
Limited joint count, median (IQR)	89	1 (1–5)
ESR, median (IQR) mm/hour	67	18 (8–43)
Physician global assessment,median (IQR) cm	64	2.5 (1.5–5.0)
Proxy global assessment of well‐being, median (IQR) cm	100	2.5 (0.9–5.2)

a Values are the number (%) unless indicated otherwise. IQR = interquartile range; ILAR = International League of Associations for Rheumatology; RF = rheumatoid factor; ESR = erythrocyte sedimentation rate.

#### Cross‐sectional comparisons between the proxy‐completed C‐HAQ, the adolescent‐specific C‐HAQ, and the HAQ.

##### Correlations and associations between overall scores

At baseline, overall score correlations were high between all pairs of measures (Table 2). However, HAQ scores (median 0.5 [IQR 0.0–1.1]) were marginally lower than either C‐HAQ score (median 0.6 [IQRs 0.1–1.3] for both; *P* < 0.001 for both compared with HAQ score). There was no significant difference between overall proxy‐completed C‐HAQ and adolescent‐specific C‐HAQ scores at baseline (*P* = 0.967). The highest correlation was between the 2 self‐completed measures, the adolescent‐specific C‐HAQ and HAQ (r = 0.91), with the lowest between the proxy‐completed C‐HAQ and adolescent‐specific C‐HAQ (r = 0.83).

**Table 2:** Baseline correlations and comparisons between the proxy‐completed C‐HAQ, the adolescent‐specific C‐HAQ, and the HAQ^a^

Comparison	Correlation	Concordant scores (≤0.25 points)	Discordant scores	
			Percent first listed higher than second listed^b^	Percent first listed lower than second listed^c^
Adolescent‐specific vs. proxy‐completed C‐HAQ scores	0.83	78	12	10
Adolescent‐specific C‐HAQ vs. HAQ scores	0.91	74	22	5
HAQ vs. proxy‐completed C‐HAQ scores	0.86	71	7	22

a Values are the percentage unless indicated otherwise. C‐HAQ = Childhood Health Assessment Questionnaire.

b Percentage increase.

c Percentage decrease.

##### Agreement between overall scores

**Figure 1:**
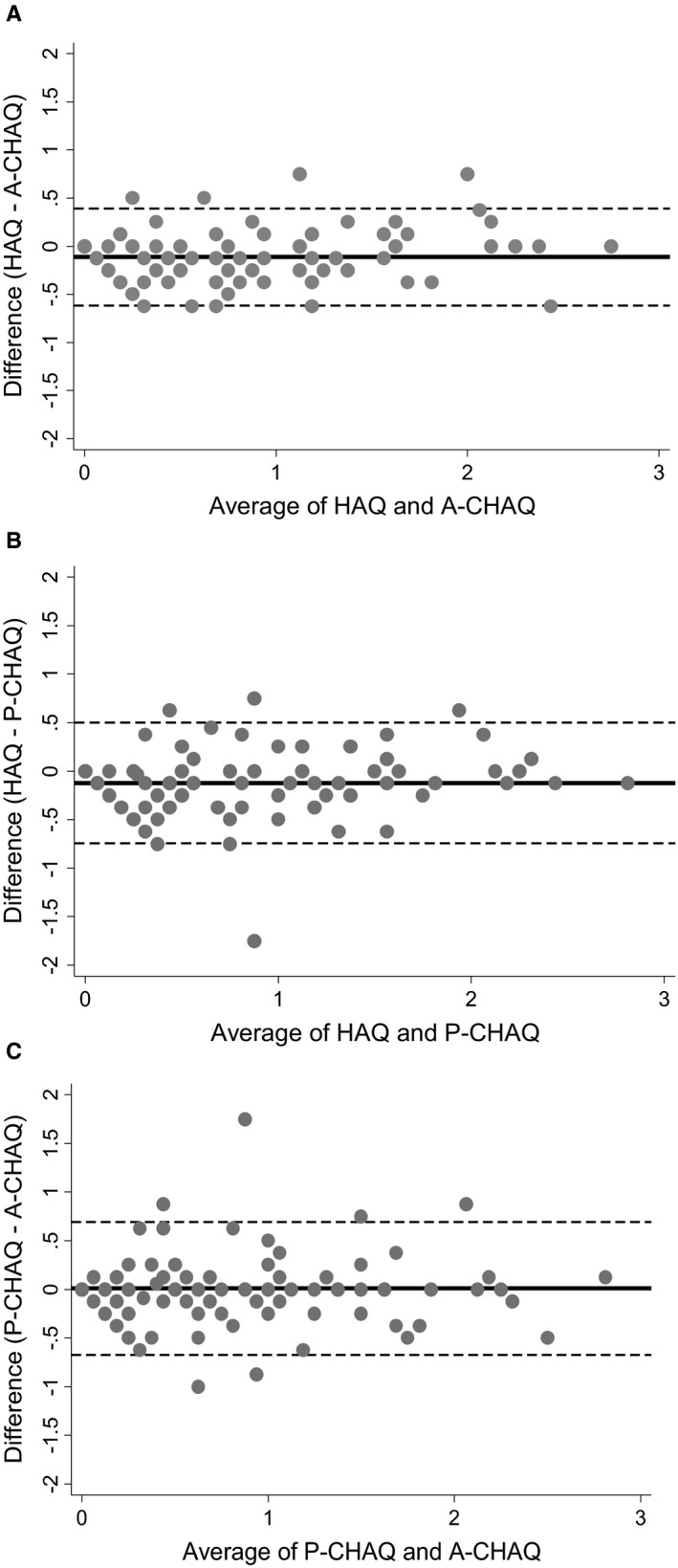
Bland‐Altman plots showing the agreement between the Health Assessment Questionnaire (HAQ) and the adolescent‐specific Childhood Health Assessment Questionnaire (A‐CHAQ) (**A**), the HAQ and the proxy‐completed CHAQ (P‐CHAQ) (**B**), and the P‐CHAQ and the A‐CHAQ (**C**). Circles represent a value where the average of 2 measures (x‐axis) and the difference between those measures (y‐axis) meet. Solid lines indicate the mean difference; broken lines indicate the 95% limits of agreement.

Good agreement was evident between all pairs of measures. Scores of zero frequently corresponded to scores of zero on opposing measures. Variation in score differences did not appear to change over the length of the scoring range (Figure 1). When assessing differences within 0.25 points 7, agreement was similar between the 3 scores with 70% to 80% for each pair (Table 2). Where discordant, the majority of HAQ scores fell below those of either C‐HAQ score. Discordance between the 2 C‐HAQ scores was more evenly distributed (Table 2).

##### Correlations between corresponding domains across measures

The vast majority of adolescents had data available on all domain‐specific scores at baseline (n = 102, 95%). Moderate to substantial agreement was evident between all pairs of scores in every domain. Particularly high agreement was evident in the eating domain, with all kappa coefficients exceeding 0.6 (Table 3). The weakest agreement was in the activities domain, with scores approximating 0.4 to 0.5 between all pairs of scores (Table 3). For this domain, the median HAQ activities score was marginally lower at 0 (IQR 0–1) compared with either C‐HAQ score (both median 1 [IQR 0–2]).

**Table 3:** Kappa coefficients between domain‐specific scores on the proxy‐completed C‐HAQ, the adolescent‐specific C‐HAQ, and the HAQ^a^

Comparison	Dressing and grooming	Arising	Eating	Walking	Hygiene	Reach	Grip	Activities
Adolescent‐specific vs. proxy‐completed C‐HAQ scores	0.51	0.53	0.66	0.59	0.65	0.56	0.61	0.47
Adolescent‐specific C‐HAQ vs. HAQ scores	0.68	0.59	0.82	0.76	0.82	0.56	0.67	0.42
HAQ vs. proxy‐completed C‐HAQ scores	0.52	0.51	0.69	0.64	0.58	0.51	0.56	0.38

a Score components were assessed after adjustment in each category for aids. C‐HAQ = Childhood Health Assessment Questionnaire.

#### Agreement in change between the proxy‐completed C‐HAQ, adolescent‐specific C‐HAQ, and the HAQ

Over the first year following initial presentation to rheumatologic care, median scores of functional ability had clinically significant improvements. At 1 year, median scores on the HAQ (median 0.0 [IQR 0.0–0.75]) clinically fell below those on the proxy‐completed HAQ (median 0.25 [IQR 0.0–1.1]) and the adolescent‐specific C‐HAQ (median 0.25 [IQR 0.0–0.9]), although they did not reach statistical significance (*P* = 0.114 for HAQ versus proxy‐completed C‐HAQ, *P* = 0.051 for HAQ versus adolescent‐specific C‐HAQ). There was no significant difference between median proxy‐completed C‐HAQ and adolescent‐specific C‐HAQ scores at 1 year (*P* = 0.144).

**Figure 2:**
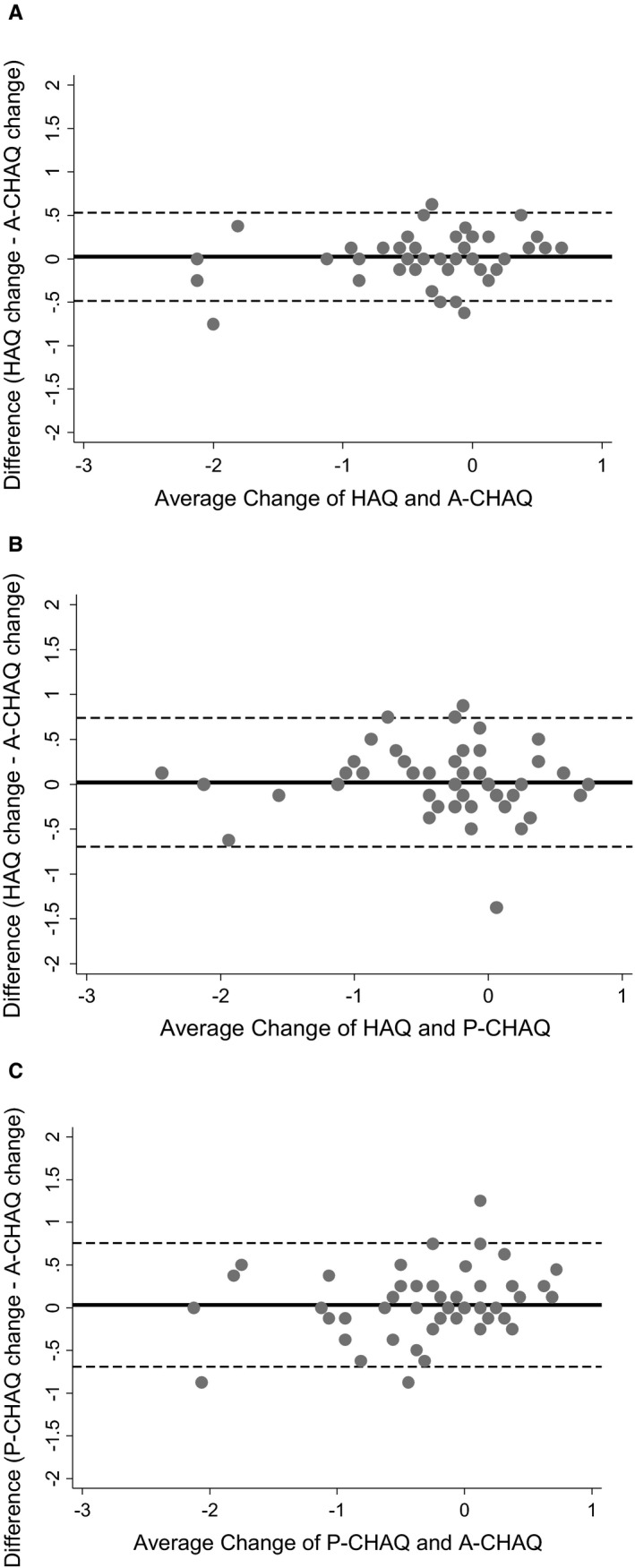
Bland‐Altman plots showing the agreement between the Health Assessment Questionnaire (HAQ) and the adolescent‐specific Childhood Health Assessment Questionnaire (A‐CHAQ) (**A**), the HAQ and the proxy‐completed CHAQ (P‐CHAQ) (**B**), and the P‐CHAQ and the A‐CHAQ (**C**) between baseline and 1 year following initial presentation to pediatric rheumatologic care. Circles represent a value where the average of 2 measures (x‐axis) and the difference between those measures (y‐axis) meet. Solid lines indicate the mean difference; broken lines indicate the 95% limits of agreement.

There was good agreement between changes in all pairs of scores, with no particular trend to change in variation over the improvement range (Figure 2). Adolescents were then classified as to whether they had experienced minimum clinically important improvements (–0.188) or worsening (+0.125) on the proxy‐completed C‐HAQ 13. The greatest ability to similarly classify these adolescents was gained through cut points of –0.5 and +0.5 on the HAQ and –0.4 and +0.125 on the adolescent‐specific C‐HAQ, respectively. However, when applying the original proxy‐completed C‐HAQ cutoffs to the respective measures, there was minimal difference (≤5%) in the percentage of adolescents identified compared with these optimum values (Table 4).

**Table 4:** Receiver operating characteristics comparing the proxy‐completed C‐HAQ with the adolescent‐specific C‐HAQ and the HAQ over the first year following initial presentation^a^

Comparison	AUC (95% CI)	Percent correctly classified under proxy‐completed C‐HAQ cutoffs^b^	Optimum cutoff	Percent correctly classified under optimum cutoff
Improvement				
Proxy‐completed vs. adolescent‐specific C‐HAQ scores	0.89 (0.77–0.95)	77, 78	–0.375	82
Proxy‐completed C‐HAQ vs. HAQscores	0.82 (0.70–0.91)	80, 80	–0.500	82
Worsening				
Proxy‐completed vs. adolescent‐specific C‐HAQ scores	0.85 (0.73–0.92)	88	+0.125	88
Proxy‐completed C‐HAQ vs. HAQscores	0.79 (0.66–0.88)	80	+0.500	82

a AUC = area under the curve; 95% CI = 95% confidence interval; C‐HAQ = Childhood Health Assessment Questionnaire.

b Proxy‐completed C‐HAQ cutoffs: –0.188 for improvement, +0.125 for worsening 13. Since an improvement of 0.188 could not be gleaned from these data, estimates are reported for –0.125 and then –0.25.

## Discussion

The 3 measures of functional ability, the proxy‐completed C‐HAQ, the adolescent‐specific C‐HAQ, and the HAQ are highly correlated and have moderate‐to‐high agreement and the ability to similarly classify change over time in a population of adolescents with JIA. However, both C‐HAQ scores consistently exceeded those from the HAQ, potentially due to differences in domain‐specific tasks, such as in the activities domain. Individual domains had moderate‐to‐substantial correlations across all 3 measures.

This study corroborates limited existing evidence suggesting that scores on the proxy‐completed C‐HAQ and adolescent‐specific C‐HAQ correlate highly, despite the former being proxy‐completed and the latter self‐completed. A previous study in this cohort assessed agreement between these 2 measures at a single point during follow‐up of 204 patients and their proxies using clinical agreement defined as ±0.25 points 7. Similar to the current study, high agreement was reported. However, adolescents included in the previous study were not all assessed at a similar time point. It was therefore unclear if agreement was high at initial presentation, when both vital first treatment decisions were made and where there are greater ranges of functional ability. This is particularly relevant in light of greater discrepancies in median HAQ and C‐HAQ scores at baseline compared with at 1 year as in the current analysis. In addition, a previous study reported greater agreement between adolescent and proxy scores on the proxy‐completed C‐HAQ, where good functional ability is evident in the context of established disease (median disease duration 5.7 years) 6. The current study shows that at initial presentation to pediatric rheumatologic care, overall scores on the proxy‐completed C‐HAQ and adolescent‐specific C‐HAQ were highly correlated and had high agreement. In addition, the changes in scores using these measures show high agreement over the first year following initial presentation and similar abilities to classify clinically important changes, with approximately 80% to 90% of adolescents who improve and worsen, respectively, over the first year of disease according to the proxy‐completed C‐HAQ similarly classified by the adolescent‐specific C‐HAQ. However, this means that there was an incorrect classification in 10% to 20% of adolescents. Thus, absolute values and changes in scores across questionnaires cannot be considered completely interchangeable.

This study is the first to compare the adolescent‐specific C‐HAQ and HAQ and can report high correlation and agreement between scores both at initial presentation and for change in scores over the first year of disease. However, as the transfer process from pediatric to adult rheumatology occurs during adolescence, the HAQ would be a preferable measure to continuously capture functional ability during this time period and beyond. For clinical trials in this age range, the HAQ would also facilitate independence of the young person in respect to their disease, where the proxy‐completed C‐HAQ does not. Existing trials and observational research in adolescents have previously used the proxy‐completed C‐HAQ and then the HAQ as young people pass through a specific age cutoff of 18 years 14, 15, 16, without adjustments or corrections for the potential differences between scores. It will therefore be unclear in these settings whether differences in scores that result when these forms are switched are due to differences in disease or outcome measure. Anecdotally, clinics recruiting to CAPS have reported returned proxy‐completed C‐HAQ forms with edits from adolescents regarding its developmental inappropriateness. As a result, we are aware that some centers are now using the HAQ at a younger age. This is likely a similar approach to that taken in other clinics in the UK. The results of this study are corroborated by limited previous studies reporting high correlations between these 2 measures 1, 17. However, the results of the current study suggest that these current practices may have been overestimating functional ability when adolescents move on to completing the HAQ. It may have seemed, therefore, that the adolescents’ function had improved when in reality their function remained stable, or their disease may have appeared to have remained stable despite worsening of function over the transition period. Van Pelt et al also reported a lack of agreement between the HAQ and a self‐reported C‐HAQ (proxy version) in 89 adolescents and young adults with chronic rheumatic disease, 76% of whom had JIA 1. They cited limits of agreement of approximately ±0.5 points as evidence of poor agreement between the measures. When clinically meaningful bounds were placed on the current study (agreement ≤0.25 points at baseline and 0.125/0.188 points for clinically important worsening/improvement, respectively, between baseline and 1 year), between 70% and 80% of adolescents were classified similarly, suggesting moderate‐to‐good discrimination between these measures. Of note, marginally more adolescents were classified similarly to the proxy‐completed C‐HAQ when cut points of ±0.5 points were used on the HAQ (82% classified correctly) instead of minimum clinically important cut points (–0.188, +0.125) on the former measure (80% classified correctly). Although previous studies have suggested a minimum clinically important difference on the HAQ in adults, with rheumatoid arthritis ranging from a 0.2‐point change to 0.5, as in the current study 18, the fact that so few adolescents were differentially classified between the proxy‐completed C‐HAQ and HAQ optimum cut points suggests that the former can be used in adolescent practice.

When focusing on domain‐specific scores, there was moderate‐to‐strong agreement between corresponding domains across measures. This corroborates evidence reported by Van Pelt et al, who estimated high intraclass correlations between corresponding domains on the self‐completed proxy‐completed C‐HAQ and the HAQ (1). In that study, the highest correlation coefficients were for the hygiene and reach domains, with the lowest for those measuring activities. The current study can corroborate higher domain‐specific agreement between both the proxy‐completed C‐HAQ and the HAQ, and the adolescent‐specific C‐HAQ and the HAQ, in the hygiene domain, with lower agreement in the activities domain. Alterations to the 2 C‐HAQ measures may have rendered this domain slightly less equivalent, with these measures asking about the ability to ride a bicycle, a form of recreation above the errands, chores (such as vacuuming, housework, or light gardening), and movement in and out of a car items captured as part of the HAQ 3, 6, 8. This discrepancy may be a driver of the lower HAQ scores compared with both C‐HAQ scores in this study. Pediatric and adolescent‐specific questionnaires may, therefore, ask questions more pertinent to adolescents with JIA, with the HAQ underestimating functional limitations during overlooked patient‐important activities.

The current study was fortunate to have over 100 adolescents and their proxies completing all 3 of the functional ability measures. These participants spanned the ILAR JIA categories and ages of adolescence. The study has highlighted clinical and statistical similarities between overall and domain‐specific scores both at initial presentation to rheumatologic care and the following year of disease, which are not identical. In clinical practice, although the adolescent‐specific C‐HAQ and HAQ were designated self‐completed and handed to young people themselves, it was not always clear whether young people had self‐completed or handed the forms to their proxies to complete as well. Previous work has identified some dissatisfaction with the C‐HAQ in young people in terms of both its length and content, which may dissuade young individuals from completing this questionnaire 19. Since proxies also had the proxy‐completed C‐HAQ to complete, this scenario was likely limited given the appointment time constraints. However, differences between questionnaire scores may be at least as large as those presented in the current study due to this potential misclassification. Slight misclassification may also have occurred where overall, rather than domain‐specific, scores were provided to the investigators. It was unclear whether aids and devices had been adjusted for in these overall scores. However, this only applied to a small number of adolescents (n = 5) and therefore was unlikely to affect the associations presented in any clinically meaningful way. An additional limitation of this study was that the 3 measures could not be compared in adolescents with severe disability. However, this inception cohort reflects the real‐world state of functional ability in the early stages of adolescent‐onset disease. That all ILAR categories were included and young people were recruited from multiple centers within the UK suggests high generalizability for these results to the general population of adolescents with JIA.

Likewise, this study investigated adolescent‐onset disease only. Since the greatest change in functional ability is observed shortly after diagnosis of JIA 20, it could be expected that adolescents with childhood‐onset JIA experience relatively mild functional disability similar to their adolescent‐onset peers, thus lending the results of this study generalizable to both groups of young individuals in adolescence. In addition, there may have been value in also comparing self‐completed C‐HAQ forms using the proxy‐completed version of the C‐HAQ. Despite the questions on this questionnaire lending themselves to proxy completion, it is common practice for young people themselves to complete the proxy‐completed C‐HAQ once over a certain age. As adolescents did not complete the proxy‐completed C‐HAQ themselves, comparisons between this and the other measures could not have been completed. However, the high correlation and agreement between the proxy‐completed C‐HAQ and self‐completed adolescent‐specific C‐HAQ suggests that adolescents and their proxies would likely not have scored dissimilarly on a single measure.

In conclusion, although the HAQ was originally considered applicable to adults, the current study reports moderate‐to‐high comparability to the adolescent‐specific C‐HAQ and the proxy‐completed C‐HAQ and thus supports its suitability for use in adolescents over the age of 10 years. However, HAQ scores may fall below C‐HAQ scores in the same young person. The absolute values of the scores are therefore not directly comparable as adolescents move from pediatric to adult practice.

## Author Contributions

All authors were involved in drafting the article or revising it critically for important intellectual content, and all authors approved the final version to be submitted for publication. Dr. Shoop‐Worrall had full access to all of the data in the study and takes responsibility for the integrity of the data and the accuracy of the data analysis.

### Study conception and design

Shoop‐Worrall, Hyrich, Verstappen, Thomson, McDonagh.

### Acquisition of data

Hyrich, Baildam, Chieng, Davidson, Foster, Ioannou, McErlane, Wedderburn, Thomson, McDonagh.

### Analysis and interpretation of data

Shoop‐Worrall, Hyrich, Verstappen, Sergeant, McDonagh.

## Additional Disclosures

Author Ioannou is an employee of UCB Celltech.

## Supporting information

Supplementary Table 1

## References

[1] Van Pelt PA , Kruize AA , Goren SS , Van Der Net J , Uiterwaal C , Kuis W , et al. Transition of rheumatologic care, from teenager to adult: which health assessment questionnaire can be best used? Clin Exp Rheumatol 2010;28:281–6. 20483054

[2] Giannini EH , Ruperto N , Ravelli A , Lovell DJ , Felson DT , Martini A . Preliminary definition of improvement in juvenile arthritis. Arthritis Rheum 1997;40:1202–9. 9214419 10.1002/1529-0131(199707)40:7<1202::AID-ART3>3.0.CO;2-R

[3] Nugent J , Grainer J , Machado C . The British version of the childhood health assessment questionnaire (CHAQ) and the child health questionnaire (CHQ). Clin Exp Rheumatol 2001;19:S163–7. 11510323

[4] Dempster H , Porepa M , Young N , Feldman BM . The clinical meaning of functional outcome scores in children with juvenile arthritis. Arthritis Rheum 2001;44:1768–74. 11508427 10.1002/1529-0131(200108)44:8<1768::AID-ART312>3.0.CO;2-Q

[5] Spiegel LR , Schneider R , Lang BA , Birdi N , Silverman ED , Laxer RM , et al. Early predictors of poor functional outcome in systemic‐onset juvenile rheumatoid arthritis: a multicenter cohort study. Arthritis Rheum 2000;43:2402–9. 11083261 10.1002/1529-0131(200011)43:11<2402::AID-ANR5>3.0.CO;2-C

[6] Shaw KL , Southwood TR , McDonagh JE , on behalf of the British Society of Paediatric and Adolescent Rheumatology, Children's Chronic Arthritis Association, Lady Hoare Trust for Physically Disabled Children, and Arthritis Care . Growing up and moving on in rheumatology: parents as proxies of adolescents with juvenile idiopathic arthritis. Arthritis Care Res (Hoboken) 2006;55:189–98. 10.1002/art.21834 16583398

[7] Lal SD , McDonagh J , Baildam E , Wedderburn LR , Gardner‐Medwin J , Foster HE , et al. Agreement between proxy and adolescent assessment of disability, pain, and well‐being in juvenile idiopathic arthritis. J Pediatr 2011;158:307–12. 20869068 10.1016/j.jpeds.2010.08.003 PMC3202630

[8] Kirwan JR , Reeback JS . Stanford Health Assessment Questionnaire modified to assess disability in British patients with rheumatoid arthritis. Br J Rheumatol 1986;25:206–9. 3708236 10.1093/rheumatology/25.2.206

[9] Adib N , Hyrich K , Thornton J , Lunt M , Davidson J , Gardner‐Medwin J , et al. Association between duration of symptoms and severity of disease at first presentation to paediatric rheumatology: results from the Childhood Arthritis Prospective Study. Rheumatology (Oxford) 2008;47:991–5. 18417527 10.1093/rheumatology/ken085 PMC2430218

[10] Bland JM , Altman DG . Statistical methods for assessing agreement between two methods of clinical measurement. Lancet 1986;1:307–10. 2868172

[11] Mukaka MM . Statistics corner: a guide to appropriate use of correlation coefficient in medical research. Malawi Med J 2012;24:69–71. 23638278 PMC3576830

[12] Altman DG . Practical statistics for medical research. London: Chapman and Hall; 1991.

[13] Brunner HI , Klein‐Gitelman MS , Miller MJ , Barron A , Baldwin N , Trombley M , et al. Minimal clinically important differences of the childhood health assessment questionnaire. J Rheumatol 2005;32:150–61. 15630741

[14] Fifi‐Mah A , Appenzeller S , Chu MY , Lupton T , Johnson N , Crawford AM , et al. Biologics use in patients with active JIA transitioning to adult clinic improves disability even after prolonged disease course [abstract]. J Rheumatol 2009;36:2570.

[15] Al‐Matar MJ , Petty RE , Tucker LB , Malleson PN , Schroeder ML , Cabral DA . The early pattern of joint involvement predicts disease progression in children with oligoarticular (pauciarticular) juvenile rheumatoid arthritis. Arthritis Rheum 2002;46:2708–15. 12384930 10.1002/art.10544

[16] Ruperto N , Levinson JE , Ravelli A , Shear ES , Link TB , Murray K , et al. Long‐term health outcomes and quality of life in American and Italian inception cohorts of patients with juvenile rheumatoid arthritis. Part I. Outcome status. J Rheumatol 1997;24:945–51. 9150087

[17] Sparsa L , Job DC , Quartier P , Kahan A , Wipff J . Quality of life of juvenile idiopathic arthritis cohort at adulthood in a transition program [abstract]. Ann Rheum Dis 2013;71:432–3.

[18] Pope JE , Khanna D , Norrie D , Ouimet JM . The minimally important difference for the health assessment questionnaire in rheumatoid arthritis clinical practice is smaller than in randomized controlled trials. J Rheumatol 2009;36:254–9. 19132791 10.3899/jrheum.080479

[19] Parsons S , Thomson W , Cresswell K , Starling B , McDonagh JE . What do young people with rheumatic disease believe to be important to research about their condition? A UK‐wide study. Pediatr Rheumatol Online J 2017;15:53. 28673355 10.1186/s12969-017-0181-1 PMC5496376

[20] Guzman J , Henrey A , Loughin T , Berard RA , Shiff NJ , Jurencak R , et al. Predicting which children with juvenile idiopathic arthritis will have a severe disease course: results from the ReACCh‐Out Cohort. J Rheumatol 2017;44:230–40. 27980015 10.3899/jrheum.160197

