# Supplementary material for: Comparing Proxy, Adolescent, and Adult Assessments of Functional Ability in Adolescents With Juvenile Idiopathic Arthritis
Source: Arthritis Care Res (Hoboken). 2020 Mar 27;72(4):517–24. doi: 10.1002/acr.23877 (PMC7154708; doi:10.1002/acr.23877)
Supplement: Supplementary file 1 — Supplementary Table 1 [file ACR-72-517-s001.docx]

**Supplementary Table 1.** Differences between the HAQ, P-CHAQ and A-CHAQ

| **Functional Ability Component** | **Original HAQ content (1)** | **Changes when developed from HAQ to P-CHAQ (2)** | **Changes when developed from P-CHAQ to A-CHAQ (3)** |
| --- | --- | --- | --- |
| Completed by | Self-completed | Proxy-completed | Self-completed |
| Outcome categories | - Without ANY difficulty - With SOME difficulty - With MUCH difficulty - UNABLE to do | Additional outcome category:   - ‘Not Applicable’ | Removal of ‘Not Applicable’ category: |
| Domain: |  |  |  |
| Dressing and grooming | 1. Dress yourself, including tying shoelaces and doing buttons 2. Shampoo your hair | Domain name changed to ‘Dressing and personal care’  Two additional categories:   1. Remove socks 2. Cut fingernails | No change |
| Rising | 1. Stand up from an armless straight chair 2. Get in and out of bed | Domain name changed to ‘Getting up’   1. ‘armless chair’ replaced by ‘low chair or floor’ | No change |
| Eating | 1. Cut your meat 2. Lift a full cup or glass to your mouth 3. Open a new carton of milk (or soap powder) | c. ‘carton of milk’ changed to ‘cereal box’ | No change |
| Walking | 1. Walk outdoors on flat ground 2. Climb up five steps | No change | No change |
| Hygiene | 1. Wash and dry your entire body 2. Take a bath 3. Get on and off the toilet | b. ‘Get in and out’ of bath added  c. ‘or potty’ added  Two additional categories:   1. Brush teeth 2. Comb/brush hair | 1. ‘or potty’ removed |
| Reach | 1. Reach and get down a 5lb object (e.g. a bag of potatoes) from just above your head 2. Bend down to pick up clothing off the floor | 1. ‘5lb object e.g. potatoes’ changed to ‘heavy object such as a large game or books’ 2. ‘or piece of paper’ added   Two additional categories:   1. Pull on a jumper over his/her head 2. Turn neck to look back over shoulder | No change |
| Grip | 1. Open car doors 2. Open jars which have been previously opened 3. Turn taps on and off | Two additional categories:  a. Write or scribble with a pen or pencil  e. Push open a door when you have to turn a door knob | a. ‘or scribble’ removed |
| Activities | 1. Run errands and shop 2. Get in and out of a car 3. Do chores such as vacuuming, housework or light gardening | b. ‘, toy car or school bus’ added  c. changed to d. and wording changed ‘wash dishes, take out rubbish, hoovering’  Additional categories:  c. Ride a bike or tricycle  e. Run | b. ‘toy car’ removed  c. ‘tricycle’ removed  d. Additional chores added ‘gardening, make bed, clean room’ |
| Aids for the first four domains | Cane, walking frame, built-up or special utensils, crutches, wheelchair, special or built-up chair, devices used specifically for dressing, other. | - ‘Cane’ changed to ‘walking stick’. - ‘Built up pencil’ added | No change |
| Aids for the final four domains | Raised toilet seat, bath seat, bath rail, long-handled appliance for reach, jar opener (for jars previously opened), other. | ‘long-handles appliance in bathroom’ added. No ‘other’ category. | No change |

**REFERENCES**

(1) Kirwan JR, Reeback JS. Stanford Health Assessment Questionnaire modified to assess disability in British patients with rheumatoid arthritis. Br J Rheumatol 1986; 25:206-9.

(2) Nugent J, Grainer J, Machado C. The British version of the childhood health assessment questionnaire (CHAQ) and the child health questionnaire (CHQ). Clin Exp Rheumatol 2001; 19:S163-7.

(3) Shaw KL, Southwood TR, McDonagh JE. Growing up and moving on in rheumatology: parents as proxies of adolescents with juvenile idiopathic arthritis. Arthritis Rheum 2006; 55:189-98.
